# That Foolish Legislature

**Published:** 1897-05

**Authors:** 


THAT FOOLISH LEGISLATURE.—In the *Texas Medical News*' reply to the protest of State Health Officer Swearingen (vide *Texas Medical News* April, 1897, and this issue of the JOURNAL) the editor (Dr. McLaughlin) says: "While I can heartily endorse the statement that Texas is great enough, strong and rich enough to take care of herself in health matters, I and many others are anxious to know if she is foolish enough to continue to bear the cost of a quarantine that is for the benefit of other States as much as it is for Texas."

Inasmuch as the "memorial" was treated with scant courtesy in the senate, and disposed of with the remark that "the State quarantine laws need no change;" and the proposition (to turn over the quarantine to the U. S. government) coming up in the House in connection with the appropriation for quarantine, was literally snowed under—defeated by a vote of 85 to 11, Dr. McLaughlin's "anxiety to know" is gratified; he is answered in the affirmative: Texas *is* "foolish" enough to maintain an effective quarantine against infectious diseases, regardless of the co-incidental protection given States beyond us. (Would it not be as wise to say that a man should go to no expense to protect his premises against burglars because it would incidentally protect premises lying beyond him perhaps?) That foolish legislature! It is remarkable that some persons are so constituted that they regard all who do not conform to their ideas and ways of thinking, as foolish. (Awhile back the doctor said *we*

were "foolish" because we honestly differed with him on some point of policy.

To that class of opinionated gentlemen the JOURNAL would commend for thoughtful consideration the fate of a certain arrogant, and conceited jelly fish:

"A jelly fish swam in a tropical sea;
And he said: This world consists solely of ME.
Now, all that I learn from the sense of touch
Is the fact of *my feelings* viewed as such;
But to think these have an external cause,
Is an inference clearly against logical laws;
Again, to suppose, as I have hitherto done,
That there are other jelly fish under the sun,
Is a poor assumption, that can't be backed
By a jot of proof, or a single fact;
In short, like Fitchte, I very much doubt
If there is anything else at all without;
And so, I've come to the plain conclusion
If the question be only set free from confusion,—
That the universe centers solely in ME,
And if I WERE NOT,—then, nothing would be.'
Just then a shark, who was passing by,
Gobbled him up in the twink of an eye,
And he died with a few convulsive twists,
But some how—the universe still exists."

Now that the question has been settled, at least for the present, it would be best, I suppose, to drop it; but we are strongly tempted to mention here an incident which will serve to show, perhaps, how some persons look at this matter. A member of the legislature said to the writer, in effect, "I do not understand the position of the Texas State Medical Associstion and its relation, either to the public health or to the quarantine service, and therefore I do not see just what they have to do with the question of cost to the State, nor why they should be concerned about it. I know of no law that recognizes that body; certainly none that connects them with the health department, however remotely. Therefore, this gratuitous advice as to the finances of the State looks a little officious. Here comes, too, gratuitous advice from another body of doctors—the Eclectics. They tell the legislature that the State is paying certain doctors too much money for their services, and suggest ways of economy which look to be not altogether disinterested. And seeing, in the matter of quarantine, that most of the expense is salaries to medical men—the officers—does it not look a little like a case of 'outs' vs. 'ins'? Why this solicitude on the part of the doctors, lest too much money be paid for services of other doctors? Can there be any suspicion of a 'bug,' do you think, Doctor?"

To all of which we replied never a word; and, like Brer Rabbit, in "Uncle Remus," we are "keepin' on aint sayin' nothin' till yit."

